# Genetics of attention deficit hyperactivity disorder

**DOI:** 10.1038/s41380-018-0070-0

**Published:** 2018-06-11

**Authors:** Stephen V. Faraone, Henrik Larsson

**Affiliations:** 1 Departments of Psychiatry and of Neuroscience and Physiology, SUNY Upstate Medical University, Syracuse, NY 13210 USA; 2 School of Medical Sciences, Örebro University, Örebro, Sweden; 3 Department of Medical Epidemiology and Biostatistics, Karolinska Institutet, Stockholm, Sweden

**Keywords:** Genetics, Neuroscience

## Abstract

Decades of research show that genes play an vital role in the etiology of attention deficit hyperactivity disorder (ADHD) and its comorbidity with other disorders. Family, twin, and adoption studies show that ADHD runs in families. ADHD’s high heritability of 74% motivated the search for ADHD susceptibility genes. Genetic linkage studies show that the effects of DNA risk variants on ADHD must, individually, be very small. Genome-wide association studies (GWAS) have implicated several genetic loci at the genome-wide level of statistical significance. These studies also show that about a third of ADHD’s heritability is due to a polygenic component comprising many common variants each having small effects. From studies of copy number variants we have also learned that the rare insertions or deletions account for part of ADHD’s heritability. These findings have implicated new biological pathways that may eventually have implications for treatment development.

Attention deficit hyperactivity disorder (ADHD) is a childhood-onset condition with impairing symptoms of inattention, impulsivity, and hyperactivity. Decades of research have documented and replicated key facts about the disorder (for a review, see ref. [1]). It occurs in about 5% of children with little geographic or cross-cultural variation in prevalence and often co-occurs with other conditions, including mood, anxiety, conduct, learning, and substance use disorders. Longitudinal studies show that two-thirds of ADHD youth will continue to have impairing symptoms of ADHD in adulthood. People with ADHD are at risk for a wide range of functional impairments: school failure, peer rejection, injuries due to accidents, criminal behavior, occupational failure, divorce, suicide, and premature death. Although many details of ADHD’s pathophysiology are unknown, neuropsychological and neuroimaging studies implicate brain circuits regulating executive functioning, reward processing, timing, and temporal information processing.

This article reviews data about the role that genes play in the etiology of ADHD from two perspectives. Family, twin, and adoption studies provide a firm foundation for asserting that genes are involved in the etiology of ADHD. The view from molecular genetics provides a basis for understanding mechanisms whereby genes affect biological pathways that lead to ADHD.

## Family, twin and adoption studies of ADHD

### Evidence for heritability from family, adoption, and twin studies

A study of 894 ADHD probands and 1135 of their siblings aged 5–17 years old found a ninefold increased risk of ADHD in siblings of ADHD probands compared with siblings of controls [2]. Adoption studies suggest that the familial factors of ADHD are attributable to genetic factors rather than shared environmental factors [3, 4] with the most recent one reporting rates of ADHD to be greater among biological relatives of non-adopted ADHD children than adoptive relatives of adopted ADHD children. The adoptive relatives had a risk for ADHD like the risk in relatives of control children [4].

**Fig. 1:**
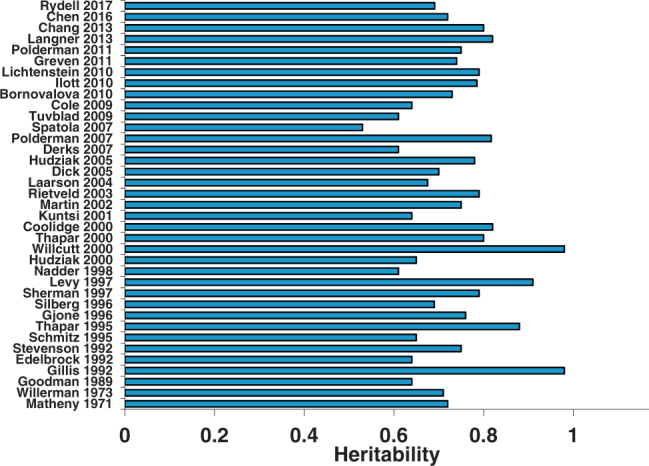
Heritability of ADHD from twin studies of ADHD diagnoses or symptom counts [153–173]

Twin studies rely on the difference between the within-pair similarities of monozygotic (MZ) twin pairs, who are genetically identical, and dizygotic (DZ) twin pairs, who share, on average, 50% of their segregating genes. The mean heritability across 37 twin studies of ADHD or measures of inattentiveness and hyperactivity is 74% (Fig. 1). A similar heritability estimate of around 80% was seen in a study of MZ and DZ twins, full siblings, and maternal and paternal half-siblings [5]. The heritability is similar in males and females and for the inattentive and hyperactive-impulsive components of ADHD [6–8].

Only a few of the twin studies in Fig. 1 used categorical measures of ADHD [9–12]. Their heritability estimates range from 77 to 88%, which is consistent with the larger number of studies using symptom count measures of ADHD. Twin studies have explored whether ADHD is best viewed as a categorical disorder or as an extreme of a continuous trait. A study of 16,366 Swedish twins found a strong genetic link between the extreme and the sub-threshold variation of DSM-IV ADHD symptoms [13]. This study confirmed an early study of 583 same-sexed twin pairs using ADHD-III-R symptoms [14]. Both studies suggest that the diagnosis of ADHD is the extreme of a continuous distribution of ADHD symptoms in the population and that the etiologic factors involved in the disorder also account for the full range of symptoms. These data are consistent with clinical studies showing the clinical implications of subthreshold ADHD [15].

### ADHD’s clinical features and course

#### Reporter effects

Parent and teacher ratings of ADHD symptoms result in high heritability estimates (70–80%) [6]. In contrast, studies using self-ratings in adolescence and adulthood show lower heritabilities (<50%) [16–19]. Two twin studies examined these rater effects [20, 21]. They showed that self-ratings, as well as different-parent and different-teacher ratings within twin pairs, were associated with lower heritability estimates (~30–40%) compared with heritabilities based on same-parent and same-teacher ratings (~70–80%) [20–22]. Low reliability of self-reports may explain why heritability estimates are lower in studies of self-rated ADHD symptoms. Using different informants for ADHD symptom ratings of each twin in a pair introduces rater effects (i.e., each rater experiences and reports different ADHD symptoms) or rater bias (i.e., a rater consistently over- or underestimates ADHD symptoms or similarities between twins). These effects could explain why heritability estimates are lower in studies relying on different informants for each twin in a pair compared with studies using the same raters [23].

#### Developmental effects

**Fig. 2:**
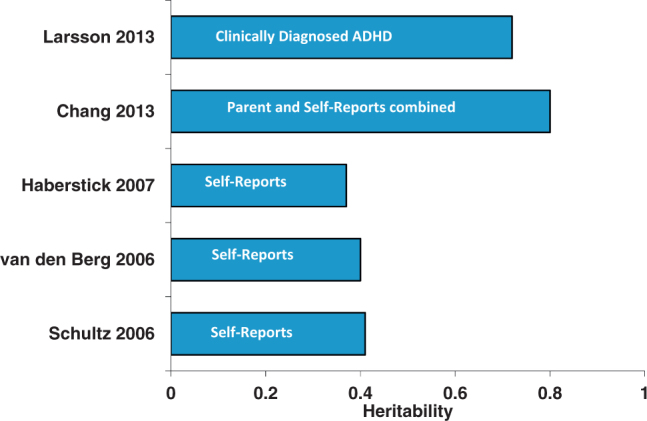
Heritability of ADHD in adults depends on method of diagnosis

The first twin studies of ADHD in adults used self-reports and estimated heritability at 30–40% (Fig. 2), (e.g. [24]), which is substantially lower than the heritability among children and adolescents. In contrast, one study estimated heritability to be 80% after combining self and parent ratings into a composite index of ADHD. Another study found the heritability of clinically diagnosed ADHD in adults to be 72% [25]. These findings (Fig. 2) suggest that the heritability of ADHD is stable during the transition from childhood into adulthood. They explain previous reports of low heritability for ADHD symptoms in adults as due to measurement error from rater effects. The higher heritabilities for clinically diagnosed adult ADHD confirm family studies suggesting that persistent ADHD is highly familial [5, 26, 27].

Twin studies show that both stable and dynamic genetic risk factors influence ADHD over the course of the development from childhood to early adulthood [7, 28–30]. These study findings explain the developmental structure of genetic risk factors for ADHD with both stable and dynamic processes. The stable component of the genetic risk suggests that persistent ADHD and its pediatric form are genetically linked. The dynamic component suggests that the set of genetic variants accounting for the onset of ADHD differs from those accounting for the persistence and remission of the disorder. For a review of the genetics of adult ADHD, see Franke et al. [26].

#### Psychiatric comorbidity

Multivariate twin and sibling studies have found a general genetic factor that influences ADHD and a broad spectrum of neuropsychiatric conditions [31, 32]. These studies have shown that a latent shared genetic factor accounts for up to 45% of co-variance across childhood externalizing, internalizing, and phobia symptoms [31, 33] and 31% of co-variance in childhood neurodevelopmental symptoms [34]. Similar results have been reported for register-based clinical diagnoses, with one study showing that a general genetic factor explained 10–36% of disorder liability across several psychiatric diagnoses [32]. Two studies have assessed the contribution of measured genetic variants for a general psychopathology dimension. One study estimated the SNP-heritability as 18% for maternal ratings of total problems on the Child Behavior Checklist, which measures internalizing, externalizing, and attention problems [35]. Similarly, another study estimated the SNP heritability as 38% for a general psychopathology factor derived from childhood psychopathology symptoms assessed by multiple raters [36]. These studies also support spectrum-specific genetic factors, such as genetic factors that load specifically on externalizing disorders [31]. The finding of externalizing-specific genetic factors for ADHD is consistent with a large number of twin and family studies demonstrating genetic overlaps of ADHD with oppositional-defiant disorder symptoms [37], conduct disorder [38], antisocial behavior [39], and substance use problems [40–42].

Twin studies have tested for genetic overlap between ADHD and autism spectrum disorders (ASD) [43, 44], which often co-occur [45]. Studies of community samples of youth, from the United States of America [46], the United Kingdom [47], and Sweden [11, 48] show that genetic factors influence this comorbidity. Ronald et al. [47]. found genetic correlations between ADHD and ASD above 0.50. Similar results have been found in adult twin studies [49]. A register-based study in Sweden found that individuals with ASD and their relatives were at increased risk of ADHD. The pattern of association across relatives supported the existence of a genetic overlap between clinically ascertained ASD and ADHD [50]. Some features of ASD are differentially linked to either the inattentive or the hyperactive-impulsive components of ADHD [51, 52]. For instance, Polderman et al. [51]. found that the symptoms reflecting the repetitive and restricted aspects of ASD showed the strongest genetic association with ADHD and a Swedish twin study found that the subcomponents of ADHD and ASD are influenced by specific genetic factors [48].

Fewer studies have explored how genetic factors contribute to the co-occurrence between ADHD and internalizing disorders. A large family study found an increased risk of attempted and completed suicide in first- and second-degree relatives of ADHD probands [53]. The pattern of familial risks across different levels of relatedness suggests that shared genetic factors are important for these associations [53]. Family studies that studied the association between ADHD and depression suggest that the co-occurrence is influenced by shared familial factors [54, 55]. Twin studies of this issue suggest that shared genetic factors explain the overlap of ADHD with depression, anxiety, and internalizing symptoms [56–60]. For example, Cole et al. [59]. found that shared genetic factors explained most of the association between traits of ADHD and depression. Similar results were found by Spatola et al. [60]., who used a multivariate twin analysis to study the overlap between different subscales of the Child Behavior Check List (CBCL), such as affective problems, anxiety problems, and attention-deficit/hyperactivity problems.

In contrast to the wealth of information about the familial co-transmission of ADHD and many other disorders, very little is known about ADHD’s familial links to intellectual disability (ID). A meta-analysis reported that the intelligence quotient (IQ) of youth with ADHD is nine points lower than typically developing peers [61] and much evidence suggests it is valid to diagnose ADHD in the context of ID [62]. Faraone et al. [63] studied the genetic association of ADHD and ID in Swedish medical registry data. Individuals with ID were at increased risk for ADHD and relatives of ID cases had an increased risk for ADHD compared with relatives of those without ID. Model fitting analyses attributed 91% of the correlation between the liabilities of ADHD and ID to genetic factors. This work attributes nearly all the comorbidity between ADHD and ID to genetic factors. Only a few twin and family studies have explored how genetic factors contribute to non-psychiatric comorbidity. The literature suggests novel etiologic links with asthma [64], obesity [65], and epilepsy [66].

## The search for common genetic variants

### Genetic linkage studies

Genetic linkage was the first genome-wide method applied to ADHD. This method searches the genome for evidence that a segment of DNA is transmitted with a disorder within families. A review of the linkage literature found substantial disagreement about which chromosomal regions are linked to ADHD [67]. Although there is some overlap in “suggestive” findings, no finding met genome-wide significance [68]. To make sense of these results, Zhou et al. [69] applied Genome Scan Meta-Analysis. They found genome-wide significant linkage for a region on chromosome 16 between 64 Mb and 83 Mb. Because the linkage method only detects genetic variants that have large effects, the paucity of significant findings for other loci suggests that common DNA variants having a large effect on ADHD are unlikely to exist. Nearly all ADHD linkage studies have selected either sibling pairs or small families from outbred populations. Another approach is to assess for linkage in multigenerational population isolates. Arcos-Burgos et al. [70] used this strategy to study 16 multi-generational families from Colombia. In some of these families, they found evidence supporting linkage to chromosomes 4q13.2, 5q33.3, 8q11.23, 11q22, and 17p11. one region implicated *LPHN3*. For a review of supporting evidence, see ref. [71].

### Candidate gene association studies

Early molecular genetic studies of ADHD sought to associate ADHD with genes that had some a priori plausibility as being involved in its etiology. Because the drugs that treat ADHD target dopaminergic or noradrenergic transmission, many studies examined “candidate genes” in these pathways. Results were frequently contradictory [26, 67]. In the meta-analyses of Gizer et al. [72], eight candidate DNA variants showed a statistically significant association with ADHD across multiple studies. These variants implicated six genes: the serotonin transporter gene (*5HTT*), the dopamine transporter gene (*DAT1*), the D4 dopamine receptor gene (*DRD4*), the D5 dopamine receptor gene (*DRD5*), the serotonin 1B receptor gene (*HTR1B*) and a gene coding for a synaptic vesicle regulating protein known as *SNAP25*. A meta-analysis covering all genetic association studies of adults with ADHD reported a significant association between adult ADHD and *BAIAP2* (brain-specific angiogenesis inhibitor 1-associated protein 2). *BAIAP2* is involved in neuronal proliferation, survival, and maturation and dendritic spine morphogenesis and may affect neuronal growth-cone guidance. These findings were significant even after Bonferroni correction [73]. For both the child and adult meta-analyses, the strength of each association, as measured by the odds ratio, is small, less than 1.5.

Many studies examined the dopamine transporter gene (*SLC6A3)*, especially a 40-base pair variable number of tandem repeats regulatory polymorphism located in the 3′-untranslated region of the gene. This variant produces two common alleles with 9- and 10-repeats (9R and 10R). In humans, the 10R allele of this polymorphism has been associated with ADHD in youth [67] while the 9R allele is associated with ADHD in adults [74]. A meta-analysis showed that the 9R allele is associated with increased DAT activity in human adults as measured by positron emission tomography [75].

### Genome-wide significant common variants

Genome-wide association studies (GWAS) scan the entire genome to detect common DNA variants having very small etiologic effects. By “common” we mean greater than 1% of the population. To do this, GWAS assay hundreds of thousands or even millions of single nucleotide polymorphisms (SNPs). Doing so has a statistical cost: to assert genome-wide statistical significance, an observed association must have a *p* value less than 0.00000005. This stringent *p* value needs very large samples.

The initial GWAS of ADHD [76–86] did not discover any DNA variants that achieved genome-wide significance, even when most of these samples were combined in meta-analysis having a sample size of 2064 trios (two parents and an ADHD child), 896 ADHD patients, and 2455 controls [87]. That study did find statistical significance for a group of candidate genes previously nominated by members of the International Multisite ADHD Genetics (IMAGE) project [88]. For a review of early GWAS studies, see Franke et al. [89]. Examination of the “molecular landscape” derived from the top findings from these initial GWAS studies along with other data concluded that genes regulating directed neurite outgrowth were strongly implicated in the etiology of ADHD [90]. Pathway and gene set analyses of GWAS data implicated pathways involved in the regulation of neurotransmitter release, neurite outgrowth and axon guidance as contributors to the etiology of ADHD [91–93].

A consortium of ADHD researchers completed a GWAS meta-analysis of 12 studies comprising 20,183 people with ADHD and 35,191 controls. For methodologic details about the studies contributing data to this meta-analysis, see Demontis et al. [94]. Twelve loci achieved genome-wide significance. None of the genome-wide significant SNPs showed significant heterogeneity between studies. Among the implicated genes, *FOXP2* is especially notable because prior work had implicated it in adult ADHD (Ribases, 2012 #26445) and in speech and language disorders [95]. A FOXP2 knockout mouse study found that the gene regulates dopamine in ADHD-associated brain regions [96].

As described by Demontis et al. [94], other genes implicated by the genome-wide significant loci have relevant biological roles. *DUSP6* regulates neurotransmitter homeostasis by affecting dopamine levels in the synapses. *SEMA6D* is expressed in the brain. It regulates neuronal wiring during embryonic development. *ST3GAL3* harbors missense mutations associated with ID. *LINC00461* is expressed in brain and includes variants associated with educational attainment. Another gene implicated at that locus is *MEF2C*, which has been associated with ID and several psychiatric disorders.

The consortium conducted several gene set analyses including three sets of genes regulated by *FOXP2*: (1) genes enriched in wild-type versus control *FOXP2* knockout mouse brains; (2) genes showing differential expression in wild-type versus *FOXP2* knockout mouse brains; and (3) genes enriched in basal ganglia or inferior frontal cortex from human fetal brain samples. None of these sets were associated with ADHD. Also, non-significant was a set of candidate genes for ADHD previously proposed by a panel of ADHD experts [88]. Among these, only *SLC9A9* showed a weak association with ADHD. No Gene Ontology gene sets attained statistical significance but a set of genes showing high intolerance to loss of function did associate with ADHD.

### Common variant ADHD as a polygenic disorder

The GWAS analyses also showed that much of ADHD’s heritability is due to the polygenic effects of many common variants each having very small effects. The SNP heritability was 0.22, which is about one-third of ADHD’s heritability computed from twin studies [97]. The polygenic architecture for ADHD was confirmed by estimating polygenic risk scores in one subset of the sample and showing that it predicted ADHD, in a dose-dependent manner, in a validation subset. As seen for other psychiatric disorders [98], the variance explained by these risk scores was low (5.5%).

Further evidence for the validity of the ADHD’s polygenic background comes from analyses showing that the relevant SNPs were enriched for annotations implicating conserved regions of the genome (which are known to have biological significance) and for regulatory elements specific to the central nervous system. The discovery of a polygenic susceptibility to ADHD does not show which DNA variants comprise the susceptibility. It does, however, support the idea that more genome-wide significant variants will be discovered in larger samples.

Martin et al. [99] showed that ADHD’s polygenic liability derived from a clinical sample predicted ASD traits in a population sample, which confirms twin study data [48, 51] and gene set analyses [100] showing genetic overlap between ADHD and ASDs. The polygenic liability score derived from Martin et al.’s ADHD case-control clinical sample also predicted both inattention and hyperactivity in the general population. This latter finding was replicated by Groen-Blokhuis et al. [101] who found that ADHD polygenic risk scores significantly predicted both parent and teacher ratings of attention in preschool- and school-aged children in the population. Likewise, Stergiakouli et al. [102] showed that the polygenic liability for ADHD traits in a population sample predicted ADHD clinical diagnoses in a case-control study. These results confirmed conclusions from twin studies that the liability for clinically defined ADHD is the extreme of a trait that varies continuously in the population [13].

Other polygenic score studies are confirming cross-disorder genetic associations previously predicted by family and twin studies. We have long known that ADHD co-occurs with conduct disorder. Both family and twin studies have implicated shared genes in this association [38, 103–105]. Consistent with this prior work, Hamshere et al. [106] reported a high polygenic risk for ADHD among children with comorbid conduct problems. In a large population study, Larsson et al. [107] reported that the relatives of ADHD individuals had an increased risk for schizophrenia and bipolar disorder. Consistent with that report, the polygenic risk score derived from a large GWAS of schizophrenia significantly discriminated ADHD cases from controls [108]. This discrimination was strongest for alleles that were risk alleles for both adult schizophrenia and adult bipolar disorder, which confirms prior family and twin data suggesting a genetic link between ADHD and bipolar disorder [109]. Moreover, a joint GWAS of ADHD and bipolar disorder reported a significant correlation between the polygenic scores of ADHD and bipolar disorder and also identified genome-wide significant loci for the two disorders [110]. Similarly, prior reports of familial co-transmission of ADHD and depression [54] have been extended by showing shared SNP heritability between the two disorders [98]. Using a novel drug challenge paradigm, Hart et al. [111] found that the polygenic scores for both schizophrenia and ADHD were associated with the euphoric response to amphetamine, which suggests that the genetic association between these disorders may be due to variants in the neural systems regulating the euphoric response to amphetamine.

**Fig. 3:**
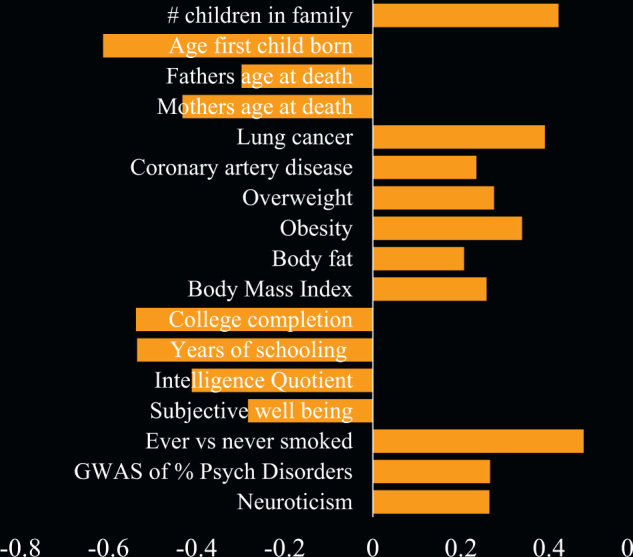
Genetic correlations of ADHD with other traits based on LD score regression

Using GWAS results from many studies, it is possible to compute genetic correlations that indicate the degree to which the polygenic architectures of two disorders or traits overlap. When Demontis et al. [94] correlated ADHD’s polygenic risk with 220 disorders and traits, many highly significant correlations emerged. Figure 3 shows some of the most significant of these correlations (each passing the Bonferroni significance threshold). Some of these genetic correlations fit with prior expectations (e.g., with neuroticism, depression and the cross disorder GWAS). Others are consistent with the clinical epidemiology of ADHD (e.g., with obesity, IQ, smoking and school achievement). In some cases, these significant correlations offer new directions for understanding comorbidity. For example, some have interpreted the comorbidity between ADHD and obesity, which has been confirmed via meta-analysis [112], as being caused by the impulsivity associated with ADHD. The genetic correlation data suggest that shared genetic risk factors, and an underlying shared pathophysiology, account for this comorbidity.

Some of the genetic correlations in Fig. 3 are entirely novel. These include ADHD’s genetic correlations with medical outcomes (lung cancer, coronary artery disease, parents’ age at death) and with demographics (number of children in the family, age first child born). There are, however, some consistent findings in the prior literature, which suggest that people with ADHD are more likely to have larger families [113] and more likely to die prematurely [114].

## The search for rare genetic variants

**Fig. 4:**
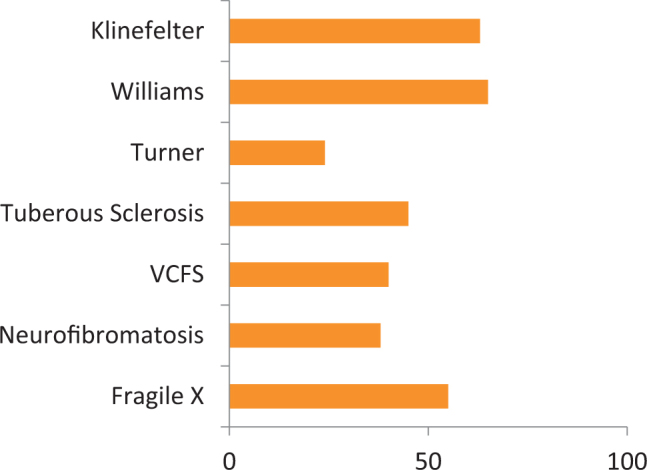
Prevalence of ADHD in rare genetic syndromes

Initially, information about rare DNA variants (<1% of the population) came from reports of syndromic chromosomal anomalies associated with multiple medical and psychiatric problems along with ADHD. Examples are velo-cardio facial syndrome fragile-X syndrome, Turner syndrome, tuberous sclerosis, neurofibromatosis, Klinefelter syndrome, and Williams syndrome (Fig. 4). In a single family, a peri-centric inversion of chromosome 3 co-segregating with ADHD symptoms [115, 116] implicated *SLC9A9*. Mutations of that gene lead to an animal model of ADHD [117, 118] and have been associated with both autism [119, 120] and ADHD [121].

The common variant genotyping arrays used in GWAS studies can detect large copy number variants (CNVs). Because CNVs often delete or duplicate a large genomic segment spanning part of a gene or even entire genes, they often have clear implications for gene functioning. Studies of CNVs in ADHD assessed ADHD youth and controls for the presence of large (>500 kb), rare CNVs [77, 122–127]. Each study, except one, found an odds ratio greater than one, indicating a greater burden of large, rare CNVs among ADHD patients compared with controls. The discrepant study used a different definition of burden [125]. Only three studies found a statistically significant burden among ADHD patients for large CNVs (for a summary, see Thapar et al. [128]). As their review shows, deletions and duplications are equally over-represented in ADHD samples although statistical significance emerged only for duplications. Thapar et al. also found enrichment for duplications (but not deletions) previously implicated in schizophrenia and, to a lesser extent, ASDs. The top biological pathways implicated by these CNV studies were: respiratory electron transport, organonitrogen compound catabolic process, transmembrane transporter activity, carbohydrate derivative catabolic process, ligand-gated ion channel activity, methyltransferase activity, transmembrane transport and ion gated channel activity.

A study of 489 ADHD patients and 1285 controls found rare CNVs in the parkinson protein two gene (*PARK2*) [123]. The result was significant after empirical correction for genome-wide testing. PARK2 regulates the cell’s ubiquitin-proteasome system which helps dispose damaged, misshapen, and excess proteins. Two other genes involved in this pathway (*FBXO33* and *RNF122*) had been implicated in other studies [84, 129]. A study of adult ADHD did not find a significant effect for large CNVs, but did find a significant effect for small CNVs [126].

The CNV studies have implicated several biological pathways. Williams et al. [127] found that ADHD patients harbored duplications in the alpha-7 nicotinic acetylcholine receptor gene (*CHRNA7)* and showed that the finding replicated in four independent cohorts from the United Kingdom, the United States, and Canada. Another replication was reported in an Italian sample [130]. The implication of the nicotinic system is particularly interesting given that nicotinic neurons modulate dopaminergic neurons, ADHD patients have a high rate of smoking [131] and nicotine administration reduces ADHD symptoms [132]. In a sample of 99 children and adolescents with severe ADHD. Lesch et al. [124] found several CNVs, including a 3 Mb duplication on chromosome 7p15.2-15.3 harboring neuropeptide Y (*NPY*). Investigation of other family members yielded an association of this duplication with increased NPY plasma concentrations and functional magnetic imaging assessed brain abnormalities.

Thapar et al. [128] reported biological pathway studies of ADHD CNV data pooled from five studies. These CNV data were enriched for genes previously implicated in schizophrenia, Fragile X intellectual disability and, to a lesser degree, autism. Several biological pathways were significantly enriched in the ADHD CNV findings, most notably ion channel pathways, which had been implicated in cross-disorder analyses of ADHD, autism, schizophrenia, bipolar disorder, and depression [133]. The CNV analyses also pointed to pathways regulating immune functioning and oxidative stress. These pathways had previously been implicated in ADHD by non-genetic studies, (e.g. refs. [134–136]).

Elia et al. [122] showed that CNVs impacting metabotropic glutamate receptor genes were significantly enriched across multiple cohorts of patients. Supporting evidence came from Akutagava-Martins et al. [137] who reported that CNVs in glutamatergic genes were associated with the cognitive and clinical impairments of ADHD. In a pharmacogenomics GWAS, an SNP in glutamate receptor gene *GRM7* was one of the most significant findings [138]. Glutamatergic defects have been observed in a rat model of ADHD [139, 140] and magnetic resonance spectroscopy in humans shows dysregulation of glutamate and glutamate/glutamine concentrations in ADHD patients(e.g. ref. [141]).

An exome sequencing study of ADHD [121] reported results for 123 adults with persistent ADHD and 82 healthy controls. Significantly more cases than controls had a rare missense or disruptive variant in a set of ADHD candidate genes. In an exome sequencing study of ADHD patients without a family history of ADHD, Kim et al. [142] reported six de novo missense SNVs in brain-expressed genes: *TBC1D9, DAGLA, QARS, CSMD2, TRPM2*, and *WDR83*. They also sequenced 26 genes implicated in ID and ASDs but found only one potentially deleterious variant. In an exome chip study, Zayats et al. [143] assayed a sample of 1846 cases and 7519 controls to search for rare genetic variants. They detected four study-wide significant loci that implicated four genes known to be expressed in the brain during prenatal stages of development: *NT5DC1*, *SEC23IP*, *PSD*, and *ZCCHC4*. Hawi et al. [144] found novel rare variants in the *BDNF* gene by sequencing 117 genes in 152 youth with ADHD and 188 controls.

## Pharmacogenetics of ADHD

Several studies have clarified the genetics of the metabolism of ADHD patients. Some patients are slow metbolizers of atomoxetine due to variants in the cytochrome P450 isoenzyme 2D6, which is regulated by the *CYP2D6* gene. As a result, the half-life of atomoxetine ranges from 5.2 h in rapid metabolizers to 21.6 h in slow metabolizers [145]. Some work has looked into CES1 variants regarding the regulation of methylphenidate metabolism and CYP2D6/CYP3A4 variants and the metabolism of ADHD, but the evidence base has not generated consistent results for either children [146] or adults [73].

Myer et al. [147] used meta-analysis to evaluate pharmacogenetic studies of the efficacy response to methylphenidate for the treatment of ADHD. They found 36 studies comprising 3647 ADHD youth treated with methylphenidate. Statistically significant effects were found for: rs1800544 in ADRA2A (odds ratio (OR): 1.69; confidence interval (CI): 1.12−2.55), rs4680 COMT (OR: 1.40; CI: 1.04−1.87), rs5569 SLC6A2 (OR: 1.73; CI: 1.26−2.37), and rs28386840 SLC6A2 (OR: 2.93; CI: 1.76−4.90), and, repeat variants VNTR 4 DRD4 (OR: 1.66; CI: 1.16−2.37) and VNTR 10 SLC6A3 (OR: 0.74; CI: 0.60−0.90). The following variants did not reach statistical significance: rs1947274 LPHN3 (OR: 0.95; CI: 0.71−1.26), rs5661665 LPHN3 (OR: 1.07; CI: 0.84−1.37) and VNTR 7 DRD4 (OR: 0.68; confidence interval: 0.47−1.00). The significant findings were not due to publication biases. Although the odds ratios are small, these findings suggest that a personalized medicine approach to ADHD is a reasonable goal of future research.

## Conclusions and future directions

There can be no doubt that DNA variants in genes or regulatory regions increase the risk for ADHD. In rare cases, a single genetic defect may lead to ADHD in the absence of other DNA variants. We do not know how many of these rare variants exist or if such variants require environmental triggers for ADHD to emerge. It is equally clear that no common DNA variants are necessary and sufficient causes of ADHD. Genome-wide association studies show that a genetic susceptibility to ADHD comprised of many common DNA variants accounts for about one-third of the twin study estimates of ADHD’s heritability. We do not know yet which variants or how many of them make up the polygenic component. The heritability that cannot be explained by main effects of rare or common variants is likely due to gene−gene interactions, gene−environment interactions or gene−environment correlations.

The convincing evidence for genes as risk factors for ADHD does not exclude the environment as a source of etiology. The fact that twin estimates of heritability are less than 100% asserts quite strongly that environmental factors must be involved. ADHD’s heritability is high, and that estimate encompasses gene by environment interaction. Thus, it is possible that such interactions will account for much of ADHD’s etiology. Environmental risk factors likely work through epigenetic mechanisms, which have barely been studied in ADHD [148]. The importance of the environment can also be seen in the fact that, as for other complex genetic disorders, much of ADHD’s heritability is explained by SNPs in regulatory regions rather than coding regions [149].

Another hypothesis for future research to explore is the possibility that ADHD is an omnigenic disorder. The omnigenic model of Boyle et al. [150] posits the existence of a small number of “core genes” having “biologically interpretable roles in disease” along with a much greater quantity of “peripheral genes” regulating the core genes. Because there are many more peripheral genes, they account for a greater proportion of the variability in heritability than do the core genes. Because core genes are more likely than peripheral genes to be relevant for developing biomarkers and treatment targets, separating these two classes from one another will require more research.

Gene discovery for ADHD has succeeded but has left us with unexpected results. None of the genome-wide significant findings had been predicted a priori and a set of ADHD candidate genes, implicated primarily by the disorder’s neuropharmacology, did not reach statistical significance. These findings challenge the idea that the core of ADHD’s pathophysiology rests within the machinery of catecholaminergic transmission. Instead, it is possible that the catecholaminergic dysregulation believed to underlie ADHD is a secondary compensation to ADHD’s primary etiology (see discussion by Hess et al. [151]).

In the years to come, we can expect breakthroughs in the genetics of ADHD to come from several fields of study. Our knowledge of rare variants should increase dramatically as we learn more about CNVs and as reports from exome, full genome and targeted sequencing studies unfold. With the discovery of genome-wide significant common variants, we look forward to studies that discover the functional variants responsible for these findings. With the discovery of these functional variants, we will learn more about the mechanisms whereby genetic risk variants increase the risk for ADHD.

Accumulating evidence from family, twin, and molecular genetic studies suggests that the disorder we know as ADHD is the extreme of a dimensional trait in the population. The dimensional nature of ADHD has wide-ranging implications. If we view ADHD as analogous to cholesterol levels, then diagnostic approaches should focus on defining the full continuum of “ADHD-traits” along with clinically meaningful thresholds for defining who does and does not need treatment and who has clinically subthreshold traits that call for careful monitoring. The dimensional nature of ADHD should also shift the debate about the increases in ADHD’s prevalence in recent years. Instead of assuming that misdiagnoses are the main explanation for the increased prevalence, perhaps researchers should explore to what extent the threshold for diagnosis has decreased over time and whether changes in the threshold are clinically sensible or not. A shift from categorical to dimensional constructs harmonizes with the Research Domain Criteria (RDoC) initiative of the National Institute of Mental Health [152]. RDoC seeks to define and validate dimensional constructs mediating psychopathology along with the neurobiological underpinnings of these constructs.

Unraveling the genetics of ADHD will be challenging. Technological advances are moving at a rapid pace. The next decade of work should give us more accurate measures of brain structure and function along with much more genomic, transcriptomic and epigenomic data. These advances will set the stage for breakthroughs in our understanding of the etiology of ADHD and in our ability to diagnose and treat the disorder.

## References

[1] Faraone SV, Asherson P, Banaschewski T, Biederman J, Buitelaar JK, Ramos-Quiroga JA (2015). Attention-deficit/hyperactivity disorder. Nat Rev Dis Prim.

[2] Chen W, Zhou K, Sham P, Franke B, Kuntsi J, Campbell D (2008). DSM-IV combined type ADHD shows familial association with sibling trait scores: a sampling strategy for QTL linkage. Am J Med Genet B Neuropsychiatr Genet.

[3] Alberts-Corush J, Firestone P, Goodman JT (1986). Attention and impulsivity characteristics of the biological and adoptive parents of hyperactive and normal control children. Am J Orthopsychiatry.

[4] Sprich S, Biederman J, Crawford MH, Mundy E, Faraone SV (2000). Adoptive and biological families of children and adolescents with ADHD. J Am Acad Child Adolesc Psychiatry.

[5] Chen Q, Brikell I, Lichtenstein P, Serlachius E, Kuja-Halkola R, Sandin S (2017). Familial aggregation of attention-deficit/hyperactivity disorder. J Child Psychol Psychiatry.

[6] Nikolas MA, Burt SA (2010). Genetic and environmental influences on ADHD symptom dimensions of inattention and hyperactivity: a meta-analysis. J Abnorm Psychol.

[7] Larsson H, Lichtenstein P, Larsson JO (2006). Genetic contributions to the development of ADHD subtypes from childhood to adolescence. J Am Acad Child Adolesc Psychiatry.

[8] McLoughlin G, Ronald A, Kuntsi J, Asherson P, Plomin R (2007). Genetic support for the dual nature of attention deficit hyperactivity disorder: substantial genetic overlap between the inattentive and hyperactive-impulsive components. J Abnorm Child Psychol.

[9] Sherman D, Iacono W, McGue M (1997). Attention deficit hyperactivity disorder dimensions: a twin study of inattention and impulsivity hyperactivity. J Am Acad Child Adolesc Psychiatry.

[10] Langner I, Garbe E, Banaschewski T, Mikolajczyk RT (2013). Twin and sibling studies using health insurance data: the example of attention deficit/hyperactivity disorder (ADHD). PLoS ONE.

[11] Lichtenstein P, Carlstrom E, Rastam M, Gillberg C, Anckarsater H (2010). The genetics of autism spectrum disorders and related neuropsychiatric disorders in childhood. Am J Psychiatry.

[12] Thapar A, Harrington R, Ross K, McGuffin P (2000). Does the definition of ADHD affect heritability?. J Am Acad Child Adolesc Psychiatry.

[13] Larsson H, Anckarsater H, Rastam M, Chang Z, Lichtenstein P (2012). Childhood attention-deficit hyperactivity disorder as an extreme of a continuous trait: a quantitative genetic study of 8,500 twin pairs. J Child Psychol Psychiatry.

[14] Levy F, Hay D, McStephen M, Wood C, Waldman I (1997). Attention-deficit hyperactivity disorder: a category or a continuum? Genetic analysis of a large-scale twin study. J Am Acad Child Adolesc Psychiatry.

[15] Faraone SV, Biederman J, Spencer T, Mick E, Murray K, Petty C (2006). Diagnosing adult attention deficit hyperactivity disorder: are late onset and subthreshold diagnoses valid?. Am J Psychiatry.

[16] Haberstick BC, Timberlake D, Hopfer CJ, Lessem JM, Ehringer MA, Hewitt JK (2008). Genetic and environmental contributions to retrospectively reported DSM-IV childhood attention deficit hyperactivity disorder. Psychol Med.

[17] Boomsma DI, Saviouk V, Hottenga JJ, Distel MA, de Moor MH, Vink JM (2010). Genetic epidemiology of attention deficit hyperactivity disorder (ADHD index) in adults. PLoS ONE.

[18] Brikell I, Kuja-Halkola R, Larsson H (2015). Heritability of attention-deficit hyperactivity disorder in adults. Am J Med Genet B Neuropsychiatr Genet.

[19] Larsson H, Asherson P, Chang Z, Ljung T, Friedrichs B, Larsson JO (2013). Genetic and environmental influences on adult attention deficit hyperactivity disorder symptoms: a large Swedish population-based study of twins. Psychol Med.

[20] Kan KJ, van Beijsterveldt CE, Bartels M, Boomsma DI (2014). Assessing genetic influences on behavior: informant and context dependency as illustrated by the analysis of attention problems. Behav Genet.

[21] Merwood A, Greven CU, Price TS, Rijsdijk F, Kuntsi J, McLoughlin G (2013). Different heritabilities but shared etiological influences for parent, teacher and self-ratings of ADHD symptoms: an adolescent twin study. Psychol Med.

[22] Kan KJ, Dolan CV, Nivard MG, Middeldorp CM, van Beijsterveldt CE, Willemsen G (2013). Genetic and environmental stability in attention problems across the lifespan: evidence from the Netherlands twin register. J Am Acad Child Adolesc Psychiatry.

[23] Freitag CM, Rohde LA, Lempp T, Romanos M (2010). Phenotypic and measurement influences on heritability estimates in childhood ADHD. Eur Child Adolesc Psychiatry.

[24] Schultz MR, Rabi K, Faraone SV, Kremen W, Lyons MJ (2006). Efficacy of retrospective recall of attention-deficit hyperactivity disorder symptoms: a twin study. Twin Res Hum Genet.

[25] Larsson H, Chang Z, D'Onofrio BM, Lichtenstein P (2014). The heritability of clinically diagnosed attention deficit hyperactivity disorder across the lifespan. Psychol Med..

[26] Franke B, Faraone SV, Asherson P, Buitelaar J, Bau CH, Ramos-Quiroga JA (2011). The genetics of attention deficit/hyperactivity disorder in adults, a review. Mol Psychiatry.

[27] Faraone SV (2004). Genetics of adult attention-deficit/hyperactivity disorder. Psychiatr Clin North Am.

[28] Chang Z, Lichtenstein P, Asherson PJ, Larsson H (2013). Developmental twin study of attention problems high heritabilities throughout development. JAMA Psychiatry.

[29] Rietveld MJ, Hudziak JJ, Bartels M, van Beijsterveldt CE, Boomsma DI (2004). Heritability of attention problems in children: longitudinal results from a study of twins, age 3 to 12. J Child Psychol Psychiatry.

[30] Kuntsi J, Rijsdijk F, Ronald A, Asherson P, Plomin R (2005). Genetic influences on the stability of attention-deficit/hyperactivity disorder symptoms from early to middle childhood. Biol Psychiatry.

[31] Lahey BB, Van Hulle CA, Singh AL, Waldman ID, Rathouz PJ (2011). Higher-order genetic and environmental structure of prevalent forms of child and adolescent psychopathology. Arch Gen Psychiatry.

[32] Pettersson E, Larsson H, Lichtenstein P (2016). Common psychiatric disorders share the same genetic origin: a multivariate sibling study of the Swedish population. Mol Psychiatry.

[33] Waldman ID, Poore HE, van Hulle C, Rathouz PJ, Lahey BB (2016). External validity of a hierarchical dimensional model of child and adolescent psychopathology: Tests using confirmatory factor analyses and multivariate behavior genetic analyses. J Abnorm Psychol.

[34] Pettersson E, Anckarsater H, Gillberg C, Lichtenstein P (2013). Different neurodevelopmental symptoms have a common genetic etiology. J Child Psychol Psychiatry.

[35] Pappa I, Fedko IO, Mileva-Seitz VR, Hottenga JJ, Bakermans-Kranenburg MJ, Bartels M (2015). Single nucleotide polymorphism heritability of behavior problems in childhood: genome-wide complex trait analysis. J Am Acad Child Adolesc Psychiatry.

[36] Neumann A, Pappa I, Lahey BB, Verhulst FC, Medina-Gomez C, Jaddoe VW (2016). Single nucleotide polymorphism heritability of a general psychopathology factor in children. J Am Acad Child Adolesc Psychiatry.

[37] Nadder TS, Rutter M, Silberg J, Maes H, Eaves L (2002). Genetic effects on the variatio and covariation of attention deficit-hyperactivity disorder (ADHD) and oppositional-defiant disorder/conduct disorder (ODD/CD) symptomatologies across informant and occasion of measurement. Psychol Med.

[38] Faraone SV, Biederman J, Monuteaux MC (2000). Attention-deficit disorder and conduct disorder in girls: evidence for a familial subtype. Biol Psychiatry.

[39] Kuja-Halkola R, Lichtenstein P, D’Onofrio BM, Larsson H (2015). Codevelopment of ADHD and externalizing behavior from childhood to adulthood. J Child Psychol Psychiatry.

[40] Chang Z, Lichtenstein P, Larsson H (2012). The effects of childhood ADHD symptoms on early-onset substance use: a Swedish twin study. J Abnorm Child Psychol.

[41] Capusan AJ, Bendtsen P, Marteinsdottir I, Kuja-Halkola R, Larsson H (2015). Genetic and environmental contributions to the association between attention deficit hyperactivity disorder and alcohol dependence in adulthood: a large population-based twin study. Am J Med Genet B Neuropsychiatr Genet.

[42] Skoglund C, Chen Q, Franck J, Lichtenstein P, Larsson H (2015). Attention-deficit/hyperactivity disorder and risk for substance use disorders in relatives. Biol Psychiatry.

[43] Rommelse NN, Geurts HM, Franke B, Buitelaar JK, Hartman CA (2011). A review on cognitive and brain endophenotypes that may be common in autism spectrum disorder and attention-deficit/hyperactivity disorder and facilitate the search for pleiotropic genes. Neurosci Biobehav Rev.

[44] Rommelse NN, Franke B, Geurts HM, Hartman CA, Buitelaar JK (2010). Shared heritability of attention-deficit/hyperactivity disorder and autism spectrum disorder. Eur Child Adolesc Psychiatry.

[45] Antshel KM, Zhang-James Y, Faraone SV (2013). The comorbidity of ADHD and autism spectrum disorder. Expert Rev Neurother.

[46] Ronald A, Edelson LR, Asherson P, Saudino KJ (2010). Exploring the relationship between autistic-like traits and ADHD behaviors in early childhood: findings from a community twin study of 2-year-olds. J Abnorm Child Psychol.

[47] Ronald A, Simonoff E, Kuntsi J, Asherson P, Plomin R (2008). Evidence for overlapping genetic influences on autistic and ADHD behaviours in a community twin sample. J Child Psychol Psychiatry.

[48] Ronald A, Larsson H, Anckarsater H, Lichtenstein P (2014). Symptoms of autism and ADHD: a Swedish twin study examining their overlap. J Abnorm Psychol.

[49] Lundstrom S, Chang Z, Kerekes N, Gumpert CH, Rastam M, Gillberg C (2011). Autistic-like traits and their association with mental health problems in two nationwide twin cohorts of children and adults. Psychol Med.

[50] Ghirardi L, Brikell I, Kuja-Halkola R, Freitag CM, Franke B, Asherson P (2017). The familial co-aggregation of ASD and ADHD: a register-based cohort study. Mol Psychiatry.

[51] Polderman TJ, Hoekstra RA, Posthuma D, Larsson H (2014). The co-occurrence of autistic and ADHD dimensions in adults: an etiological study in 17,770 twins. Transl Psychiatry.

[52] Polderman TJ, Hoekstra RA, Vinkhuyzen AA, Sullivan PF, van der Sluis S, Posthuma D (2013). Attentional switching forms a genetic link between attention problems and autistic traits in adults. Psychol Med.

[53] Ljung T, Chen Q, Lichtenstein P, Larsson H (2014). Common etiological factors of attention-deficit/hyperactivity disorder and suicidal behavior: a population-based study in Sweden. JAMA Psychiatry.

[54] Faraone SV, Biederman J (1997). Do attention deficit hyperactivity disorder and major depression share familial risk factors?. J Nerv Ment Dis.

[55] Faraone SV, Biederman J (1998). Depression: a family affair. Lancet.

[56] Rydell M, Taylor MJ, Larsson H (2017). Genetic and environmental contributions to the association between ADHD and affective problems in early childhood-A Swedish population-based twin study. Am J Med Genet B Neuropsychiatr Genet..

[57] Segenreich D, Paez MS, Regalla MA, Fortes D, Faraone SV, Sergeant J (2015). Multilevel analysis of ADHD, anxiety and depression symptoms aggregation in families. Eur Child Adolesc Psychiatry.

[58] Chen TJ, Ji CY, Wang SS, Lichtenstein P, Larsson H, Chang Z (2016). Genetic and environmental influences on the relationship between ADHD symptoms and internalizing problems: A Chinese twin study. Am J Med Genet B Neuropsychiatr Genet.

[59] Cole J, Ball HA, Martin NC, Scourfield J, McGuffin P (2009). Genetic overlap between measures of hyperactivity/inattention and mood in children and adolescents. J Am Acad Child Adolesc Psychiatry.

[60] Spatola CA, Fagnani C, Pesenti-Gritti P, Ogliari A, Stazi MA, Battaglia M (2007). A general population twin study of the CBCL/6-18 DSM-oriented scales. J Am Acad Child Adolesc Psychiatry.

[61] Frazier TW, Demaree HA, Youngstrom EA (2004). Meta-analysis of intellectual and neuropsychological test performance in attention-deficit/hyperactivity disorder. Neuropsychology.

[62] Antshel KM, Phillips MH, Gordon M, Barkley R, Faraone SV (2006). Is ADHD a valid disorder in children with intellectual delays?. Clin Psychol Rev.

[63] Faraone SV, Ghirardi L, Kuja-Halkola R, Lichtenstein P, Larsson H (2017). The familial co-aggregation of attention-deficit/hyperactivity disorder and intellectual disability: a register-based family study. J Am Acad Child Adolesc Psychiatry.

[64] Mogensen N, Larsson H, Lundholm C, Almqvist C (2011). Association between childhood asthma and ADHD symptoms in adolescence—a prospective population-based twin study. Allergy.

[65] Chen Q, Kuja-Halkola R, Sjolander A, Serlachius E, Cortese S, Faraone SV (2017). Shared familial risk factors between attention-deficit/hyperactivity disorder and overweight/obesity—a population-based familial coaggregation study in Sweden. J Child Psychol Psychiatry.

[66] Brikell I, Ghirardi L, D’Onofrio BM, Dunn DW, Almqvist C, Dalsgaard S (2018). Familial liability to epilepsy and attention-deficit/hyperactivity disorder: a nationwide cohort study. Biol Psychiatry.

[67] Faraone SV, Mick E (2010). Molecular genetics of attention deficit hyperactivity disorder. Psychiatr Clin North Am.

[68] Lander E, Kruglyak L (1995). Genetic dissection of complex traits: guidelines for interpreting and reporting linkage results. Nat Genet.

[69] Zhou K, Dempfle A, Arcos-Burgos M, Bakker SC, Banaschewski T, Biederman J (2008). Meta-analysis of genome-wide linkage scans of attention deficit hyperactivity disorder. Am J Med Genet B Neuropsychiatr Genet.

[70] Arcos-Burgos M, Castellanos FX, Pineda D, Lopera F, David Palacio J, Guillermo Palacio L (2004). Attention-deficit/hyperactivity disorder in a population isolate: linkage to Loci at 4q13.2, 5q33.3, 11q22, and 17p11. Am J Hum Genet.

[71] Arcos-Burgos M, Muenke M (2010). Toward a better understanding of ADHD: LPHN3 gene variants and the susceptibility to develop ADHD. Atten Defic Hyperact Disord.

[72] Gizer IR, Ficks C, Waldman ID (2009). Candidate gene studies of ADHD: a meta-analytic review. Hum Genet.

[73] Bonvicini C, Faraone SV, Scassellati C (2016). Attention-deficit hyperactivity disorder in adults: a systematic review and meta-analysis of genetic, pharmacogenetic and biochemical studies. Mol Psychiatry.

[74] Franke B, Vasquez AA, Johansson S, Hoogman M, Romanos J, Boreatti-Hummer A (2010). Multicenter analysis of the SLC6A3/DAT1 VNTR haplotype in persistent ADHD suggests differential involvement of the gene in childhood and persistent ADHD. Neuropsychopharmacology.

[75] Faraone SV, Spencer TJ, Madras BK, Zhang-James Y, Biederman J (2014). Functional effects of dopamine transporter gene genotypes on in vivo dopamine transporter functioning: a meta-analysis. Mol Psychiatry.

[76] Neale BM, Medland S, Ripke S, Anney RJ, Asherson P, Buitelaar J (2010). Case-control genome-wide association study of attention-deficit/hyperactivity disorder. J Am Acad Child Adolesc Psychiatry.

[77] Yang L, Neale BM, Liu L, Lee SH, Wray NR, Ji N (2013). Polygenic transmission and complex neuro developmental network for attention deficit hyperactivity disorder: genome-wide association study of both common and rare variants. Am J Med Genet B Neuropsychiatr Genet.

[78] Ebejer JL, Duffy DL, van der Werf J, Wright MJ, Montgomery G, Gillespie NA (2013). Genome-wide association study of inattention and hyperactivity-impulsivity measured as quantitative traits. Twin Res Hum Genet.

[79] Fliers EA, Vasquez AA, Poelmans G, Rommelse N, Altink M, Buschgens C (2012). Genome-wide association study of motor coordination problems in ADHD identifies genes for brain and muscle function. World J Biol Psychiatry.

[80] Hinney A, Scherag A, Jarick I, Albayrak O, Putter C, Pechlivanis S (2011). Genome-wide association study in German patients with attention deficit/hyperactivity disorder. Am J Med Genet B Neuropsychiatr Genet.

[81] Lasky-Su J, Anney RJ, Neale BM, Franke B, Zhou K, Maller JB (2008). Genome-wide association scan of the time to onset of attention deficit hyperactivity disorder. Am J Med Genet B Neuropsychiatr Genet.

[82] Lasky-Su J, Neale BM, Franke B, Anney RJ, Zhou K, Maller JB (2008). Genome-wide association scan of quantitative traits for attention deficit hyperactivity disorder identifies novel associations and confirms candidate gene associations. Am J Med Genet B Neuropsychiatr Genet.

[83] Mick E, Todorov A, Smalley S, Hu X, Loo S, Todd RD (2010). Family-based genome-wide association scan of attention-deficit/hyperactivity disorder. J Am Acad Child Adolesc Psychiatry.

[84] Sanchez-Mora C, Ramos-Quiroga JA, Bosch R, Corrales M, Garcia-Martinez I, Nogueira M (2015). Case-control genome-wide association study of persistent attention-deficit hyperactivity disorder identifies FBXO33 as a novel susceptibility gene for the disorder. Neuropsychopharmacology.

[85] Lesch KP, Timmesfeld N, Renner TJ, Halperin R, Roser C, Nguyen TT (2008). Molecular genetics of adult ADHD: converging evidence from genome-wide association and extended pedigree linkage studies. J Neural Transm.

[86] Zayats T, Athanasiu L, Sonderby I, Djurovic S, Westlye LT, Tamnes CK (2015). Genome-wide analysis of attention deficit hyperactivity disorder in Norway. PLoS ONE.

[87] Neale BM, Medland SE, Ripke S, Asherson P, Franke B, Lesch KP (2010). Meta-analysis of genome-wide association studies of attention-deficit/hyperactivity disorder. J Am Acad Child Adolesc Psychiatry.

[88] Brookes K, Xu X, Chen W, Zhou K, Neale B, Lowe N (2006). The analysis of 51 genes in DSM-IV combined type attention deficit hyperactivity disorder: association signals in DRD4, DAT1 and 16 other genes. Mol Psychiatry.

[89] Franke B, Neale BM, Faraone SV (2009). Genome-wide association studies in ADHD. Hum Genet.

[90] Poelmans G, Pauls DL, Buitelaar JK, Franke B (2011). Integrated genome-wide association study findings: identification of a neurodevelopmental network for attention deficit hyperactivity disorder. Am J Psychiatry.

[91] Mooney MA, McWeeney SK, Faraone SV, Hinney A, Hebebrand J, Consortium I (2016). Pathway analysis in attention deficit hyperactivity disorder: an ensemble approach. Am J Med Genet B Neuropsychiatr Genet.

[92] Aebi M, van Donkelaar MM, Poelmans G, Buitelaar JK, Sonuga-Barke EJ, Stringaris A (2016). Gene-set and multivariate genome-wide association analysis of oppositional defiant behavior subtypes in attention-deficit/hyperactivity disorder. Am J Med Genet B Neuropsychiatr Genet.

[93] Groenman AP, Greven CU, van Donkelaar MM, Schellekens A, van Hulzen KJ, Rommelse N (2016). Dopamine and serotonin genetic risk scores predicting substance and nicotine use in attention deficit/hyperactivity disorder. Addict Biol.

[94] Demontis D, Walters RK, Martin J, Mattheisen M, Als TD, Agerbo E (2017). Discovery of the first genome-wide significant risk loci for ADHD. Submitted for publication. bioRxiv..

[95] Lai CS, Gerrelli D, Monaco AP, Fisher SE, Copp AJ (2003). FOXP2 expression during brain development coincides with adult sites of pathology in a severe speech and language disorder. Brain.

[96] Enard W, Gehre S, Hammerschmidt K, Holter SM, Blass T, Somel M (2009). A humanized version of Foxp2 affects cortico-basal ganglia circuits in mice. Cell.

[97] Faraone SV, Perlis RH, Doyle AE, Smoller JW, Goralnick JJ, Holmgren MA (2005). Molecular genetics of attention-deficit/hyperactivity disorder. Biol Psychiatry.

[98] Lee SH, Ripke S, Neale BM, Faraone SV, Purcell SM, Perlis RH (2013). Genetic relationship between five psychiatric disorders estimated from genome-wide SNPs. Nat Genet.

[99] Martin J, Hamshere ML, Stergiakouli E, O’Donovan MC, Thapar A (2014). Genetic risk for attention-deficit/hyperactivity disorder contributes to neurodevelopmental traits in the general population. Biol Psychiatry.

[100] Bralten J, van Hulzen KJ, Martens MB, Galesloot TE, Arias Vasquez A, Kiemeney LA, et al. Autism spectrum disorders and autistic traits share genetics and biology. Mol Psychiatry. 2017;00:1–8. 10.1038/mp.2017.127 28630455

[101] Groen-Blokhuis MM, Middeldorp CM, Kan KJ, Abdellaoui A, van Beijsterveldt CE, Ehli EA (2014). Attention-deficit/hyperactivity disorder polygenic risk scores predict attention problems in a population-based sample of children. J Am Acad Child Adolesc Psychiatry.

[102] Stergiakouli E, Martin J, Hamshere ML, Langley K, Evans DM, St Pourcain B (2015). Shared genetic influences between attention-deficit/hyperactivity disorder (ADHD) traits in children and clinical ADHD. J Am Acad Child Adolesc Psychiatry.

[103] Faraone S, Biederman J, Garcia Jetton J, Tsuang M (1997). Attention deficit disorder and conduct disorder: longitudinal evidence for a familial subtype. Psychol Med.

[104] Faraone SV, Biederman J, Keenan K, Tsuang MT (1991). Separation of DSM-III attention deficit disorder and conduct disorder: evidence from a family-genetic study of American child psychiatric patients. Psychol Med.

[105] Thapar A, Harrington R, McGuffin P (2001). Examining the comorbidity of ADHD-related behaviours and conduct problems using a twin study design. Br J Psychiatry.

[106] Hamshere ML, Langley K, Martin J, Agha SS, Stergiakouli E, Anney RJ (2013). High loading of polygenic risk for ADHD in children with comorbid aggression. Am J Psychiatry.

[107] Larsson H, Ryden E, Boman M, Langstrom N, Lichtenstein P, Landen M (2013). Risk of bipolar disorder and schizophrenia in relatives of people with attention-deficit hyperactivity disorder. Br J Psychiatry.

[108] Hamshere ML, Stergiakouli E, Langley K, Martin J, Holmans P, Kent L (2013). Shared polygenic contribution between childhood attention-deficit hyperactivity disorder and adult schizophrenia. Br J Psychiatry.

[109] Faraone SV, Biederman J, Wozniak J (2012). Examining the comorbidity between attention deficit hyperactivity disorder and bipolar I disorder: a meta-analysis of family genetic studies. Am J Psychiatry.

[110] van Hulzen KJE, Scholz CJ, Franke B, Ripke S, Klein M, McQuillin A (2017). Genetic overlap between attention-deficit/hyperactivity disorder and bipolar disorder: evidence from genome-wide association study meta-analysis. Biol Psychiatry.

[111] Hart AB, Gamazon ER, Engelhardt BE, Sklar P, Kahler AK, Hultman CM (2014). Genetic variation associated with euphorigenic effects of d-amphetamine is associated with diminished risk for schizophrenia and attention deficit hyperactivity disorder. Proc Natl Acad Sci USA.

[112] Cortese S, Moreira-Maia CR, St Fleur D, Morcillo-Penalver C, Rohde LA, Faraone SV (2015). Association between ADHD and obesity: a systematic review and meta-analysis. Am J Psychiatry.

[113] Barkley RA, Murphy KR, Fischer M (2010). ADHD in Adults: What the Science Says.

[114] Dalsgaard S, Ostergaard SD, Leckman JF, Mortensen PB, Pedersen MG (2015). Mortality in children, adolescents, and adults with attention deficit hyperactivity disorder: a nationwide cohort study. Lancet.

[115] Lo-Castro A, D’Agati E, Curatolo P (2010). ADHD and genetic syndromes. Brain Dev.

[116] de Silva MG, Elliott K, Dahl HH, Fitzpatrick E, Wilcox S, Delatycki M (2003). Disruption of a novel member of a sodium/hydrogen exchanger family and DOCK3 is associated with an attention deficit hyperactivity disorder-like phenotype. J Med Genet.

[117] Zhang-James Y, Middleton FA, Sagvolden T, Faraone SV (2012). Differential expression of SLC9A9 and interacting molecules in the hippocampus of rat models for attention deficit/hyperactivity disorder. Dev Neurosci.

[118] Zhang-James Y, Dasbanerjee T, Sagvolden T, Middleton FA, Faraone SV (2011). SLC9A9 mutations, gene expression, and protein−protein interactions in rat models of attention-deficit/hyperactivity disorder. Am J Med Genet B Neuropsychiatr Genet.

[119] Patak J, Hess JL, Zhang-James Y, Glatt SJ (2016). Faraone SV SLC9A9 Co-expression modules in autism-associated brain regions. Autism Res..

[120] Patak J, Zhang-James Y, Faraone SV (2016). Endosomal system genetics and autism spectrum disorders: a literature review. Neurosci Biobehav Rev.

[121] Demontis D, Lescai F, Borglum A, Glerup S, Ostergaard SD, Mors O (2016). Whole-exome sequencing reveals increased burden of rare functional and disruptive variants in candidate risk genes in individuals with persistent attention-deficit/hyperactivity disorder. J Am Acad Child Adolesc Psychiatry.

[122] Elia J, Glessner JT, Wang K, Takahashi N, Shtir CJ, Hadley D (2012). Genome-wide copy number variation study associates metabotropic glutamate receptor gene networks with attention deficit hyperactivity disorder. Nat Genet.

[123] Jarick I, Volckmar AL, Putter C, Pechlivanis S, Nguyen TT, Dauvermann MR (2012). Genome-wide analysis of rare copy number variations reveals PARK2 as a candidate gene for attention-deficit/hyperactivity disorder. Mol Psychiatry.

[124] Lesch KP, Selch S, Renner TJ, Jacob C, Nguyen TT, Hahn T (2011). Genome-wide copy number variation analysis in attention-deficit/hyperactivity disorder: association with neuropeptide Y gene dosage in an extended pedigree. Mol Psychiatry.

[125] Lionel AC, Crosbie J, Barbosa N, Goodale T, Thiruvahindrapuram B, Rickaby J (2011). Rare copy number variation discovery and cross-disorder comparisons identify risk genes for ADHD. Sci Transl Med.

[126] Ramos-Quiroga JA, Sanchez-Mora C, Casas M, Garcia-Martinez I, Bosch R, Nogueira M (2014). Genome-wide copy number variation analysis in adult attention-deficit and hyperactivity disorder. J Psychiatr Res.

[127] Williams NM, Franke B, Mick E, Anney RJ, Freitag CM, Gill M (2012). Genome-wide analysis of copy number variants in attention deficit/hyperactivity disorder confirms the role of rare variants and implicates duplications at 15q13.3. Am J Psychiatry.

[128] Thapar A, Martin J, Mick E, Arias Vasquez A, Langley K, Scherer SW (2015). Psychiatric gene discoveries shape evidence on ADHD’s biology. Mol Psychiatry.

[129] Garcia-Martinez I, Sanchez-Mora C, Soler Artigas M, Rovira P, Pagerols M, Corrales M (2017). Gene-wide association study reveals RNF122 ubiquitin ligase as a novel susceptibility gene for attention deficit hyperactivity disorder. Sci Rep.

[130] Valbonesi S, Magri C, Traversa M, Faraone SV, Cattaneo A, Milanesi E (2015). Copy number variants in attention-deficit hyperactive disorder: identification of the 15q13 deletion and its functional role. Psychiatr Genet.

[131] Kollins SH, McClernon FJ, Fuemmeler BF (2005). Association between smoking and attention-deficit/hyperactivity disorder symptoms in a population-based sample of young adults. Arch Gen Psychiatry.

[132] Levin ED, Conners CK, Silva D, Hinton SC, Meck WH, March J (1998). Transdermal nicotine effects on attention. Psychopharmacol (Berl).

[133] Cross-Disorder Group of the Psychiatric Genomics Consortium. (2013). Identification of risk loci with shared effects on five major psychiatric disorders: a genome-wide analysis. Lancet.

[134] Buske-Kirschbaum A, Schmitt J, Plessow F, Romanos M, Weidinger S, Roessner V (2013). Psychoendocrine and psychoneuroimmunological mechanisms in the comorbidity of atopic eczema and attention deficit/hyperactivity disorder. Psychoneuroendocrinology.

[135] Joseph N, Zhang-James Y, Perl A, Faraone SV (2015). Oxidative stress and attention deficit hyperactivity disorder: a meta-analysis. J Atten Disord.

[136] Chen MH, Su TP, Chen YS, Hsu JW, Huang KL, Chang WH (2013). Comorbidity of allergic and autoimmune diseases among patients with ADHD: a nationwide population-based study. J Atten Disord.

[137] Akutagava-Martins GC, Salatino-Oliveira A, Genro JP, Contini V, Polanczyk G, Zeni C (2014). Glutamatergic copy number variants and their role in attention-deficit/hyperactivity disorder. Am J Med Genet B Neuropsychiatr Genet.

[138] Mick E, Neale B, Middleton FA, McGough JJ, Faraone SV (2008). Genome-wide association study of response to methylphenidate in 187 children with attention-deficit/hyperactivity disorder. Am J Med Genet B Neuropsychiatr Genet.

[139] Russell VA (2003). Dopamine hypofunction possibly results from a defect in glutamate-stimulated release of dopamine in the nucleus accumbens shell of a rat model for attention deficit hyperactivity disorder--the spontaneously hypertensive rat. Neurosci Biobehav Rev.

[140] Jensen V, Rinholm JE, Johansen TJ, Medin T, Storm-Mathisen J, Sagvolden T (2009). *N*-methyl-d-aspartate receptor subunit dysfunction at hippocampal glutamatergic synapses in an animal model of attention-deficit/hyperactivity disorder. Neuroscience.

[141] Carrey NJ, MacMaster FP, Gaudet L, Schmidt MH (2007). Striatal creatine and glutamate/glutamine in attention-deficit/hyperactivity disorder. J Child Adolesc Psychopharmacol.

[142] Kim DS, Burt AA, Ranchalis JE, Wilmot B, Smith JD, Patterson KE (2017). Sequencing of sporadic Attention-Deficit Hyperactivity Disorder (ADHD) identifies novel and potentially pathogenic de novo variants and excludes overlap with genes associated with autism spectrum disorder. Am J Med Genet B Neuropsychiatr Genet.

[143] Zayats T, Jacobsen KK, Kleppe R, Jacob CP, Kittel-Schneider S, Ribases M (2016). Exome chip analyses in adult attention deficit hyperactivity disorder. Transl Psychiatry.

[144] Hawi Z, Cummins TD, Tong J, Arcos-Burgos M, Zhao Q, Matthews N (2017). Rare DNA variants in the brain-derived neurotrophic factor gene increase risk for attention-deficit hyperactivity disorder: a next-generation sequencing study. Mol Psychiatry.

[145] Trzepacz PT, Williams DW, Feldman PD, Wrishko RE, Witcher JW, Buitelaar JK (2008). CYP2D6 metabolizer status and atomoxetine dosing in children and adolescents with ADHD. Eur Neuropsychopharmacol.

[146] Froehlich TE, McGough JJ, Stein MA (2010). Progress and promise of attention-deficit hyperactivity disorder pharmacogenetics. CNS Drugs.

[147] Myer NM, Boland JR, Faraone SV. Pharmacogenetics predictors of methylphenidate efficacy in childhood ADHD. Mol Psychiatry. 2017;00:1–8. 10.1038/mp.2017.234 PMC7039663 29230023

[148] Walton E, Pingault JB, Cecil CA, Gaunt TR, Relton CL, Mill J (2016). Epigenetic profiling of ADHD symptoms trajectories: a prospective, methylome-wide study. Mol Psychiatry.

[149] Gusev A, Lee SH, Trynka G, Finucane H, Vilhjalmsson BJ, Xu H (2014). Partitioning heritability of regulatory and cell-type-specific variants across 11 common diseases. Am J Hum Genet.

[150] Boyle EA, Li YI, Pritchard JK (2017). An expanded view of complex traits: from polygenic to omnigenic. Cell.

[151] Hess JL, Akutagava-Martins GC, Patak JD, Glatt SJ, Faraone SV. Why is there selective subcortical vulnerability in ADHD? Clues from postmortem brain gene expression data. Molecular Psychiatry, 00:1–7. 10.1038/mp.2017.242 PMC6985986 29180674

[152] Sanislow CA, Pine DS, Quinn KJ, Kozak MJ, Garvey MA, Heinssen RK (2010). Developing constructs for psychopathology research: research domain criteria. J Abnorm Psychol.

[153] Chang Z, Lichtenstein P, Asherson PJ, Larsson H (2013). Developmental twin study of attention problems: high heritabilities throughout development. JAMA Psychiatry.

[154] Polderman TJ, Huizink AC, Verhulst FC, van Beijsterveldt CE, Boomsma DI, Bartels M (2011). A genetic study on attention problems and academic skills: results of a longitudinal study in twins. J Can Acad Child Adolesc Psychiatry.

[155] Greven CU, Rijsdijk FV, Plomin R (2011). A twin study of ADHD symptoms in early adolescence: hyperactivity-impulsivity and inattentiveness show substantial genetic overlap but also genetic specificity. J Abnorm Child Psychol.

[156] Ilott N, Saudino KJ, Wood A, Asherson P (2010). A genetic study of ADHD and activity level in infancy. Genes Brain Behav.

[157] Bornovalova MA, Hicks BM, Iacono WG, McGue M (2010). Familial transmission and heritability of childhood disruptive disorders. Am J Psychiatry.

[158] Tuvblad C, Zheng M, Raine A, Baker LA (2009). A common genetic factor explains the covariation among ADHD ODD and CD symptoms in 9−10 year old boys and girls. J Abnorm Child Psychol.

[159] Polderman TJ, Derks EM, Hudziak JJ, Verhulst FC, Posthuma D, Boomsma DI (2007). Across the continuum of attention skills: a twin study of the SWAN ADHD rating scale. J Child Psychol Psychiatry.

[160] Derks EM, Dolan CV, Hudziak JJ, Neale MC, Boomsma DI (2007). Assessment and etiology of attention deficit hyperactivity disorder and oppositional defiant disorder in boys and girls. Behav Genet.

[161] Hudziak JJ, Derks EM, Althoff RR, Rettew DC, Boomsma DI (2005). The genetic and environmental contributions to attention deficit hyperactivity disorder as measured by the conners’ rating scales—revised. Am J Psychiatry.

[162] Dick DM, Viken RJ, Kaprio J, Pulkkinen L, Rose RJ (2005). Understanding the covariation among childhood externalizing symptoms: genetic and environmental influences on conduct disorder, attention deficit hyperactivity disorder, and oppositional defiant disorder symptoms. J Abnorm Child Psychol.

[163] Larsson JO, Larsson H, Lichtenstein P (2004). Genetic and environmental contributions to stability and change of ADHD symptoms between 8 and 13 years of age: a longitudinal twin study. J Am Acad Child Adolesc Psychiatry.

[164] Martin N, Scourfield J, McGuffin P (2002). Observer effects and heritability of childhood attention-deficit hyperactivity disorder symptoms. Br J Psychiatry.

[165] Kuntsi J, Stevenson J (2001). Psychological mechanisms in hyperactivity: II The role of genetic factors. J Child Psychol Psychiatry.

[166] Coolidge FL, Thede LL, Young SE (2000). Heritability and the comorbidity of attention deficit hyperactivity disorder with behavioral disorders and executive function deficits: a preliminary investigation. Dev Neuropsychol.

[167] Schmitz S, Fulker DW, Mrazek DA (1995). Problem behavior in early and middle childhood: an initial behavior genetic analysis. J Child Psychol Psychiatry.

[168] Stevenson J (1992). Evidence for a genetic etiology in hyperactivity in children. Behav Genet.

[169] Edelbrock C, Rende R, Plomin R, Thompson LA (1995). A twin study of competence and problem behavior in childhood and early adolescence. J Child Psychol Psychiat.

[170] Gillis JJ, Gilger JW, Pennington BF, DeFries JC (1992). Attention deficit disorder in reading-disabled twins: evidence for a genetic etiology. J Abnorm Child Psychol.

[171] Goodman R (1989). Genetic factors in hyperactivity: account for about half of the explainable variance. Br Med J.

[172] Willerman L (1973). Activity level and hyperactivity in twins. Child Dev.

[173] Matheny AP, Brown AM (1971). Activity, motor coordination and attention: individual differences in twins. Percept Mot Skills.

